# Supplementary material for: The MTNR1B rs10830963 Variant in Interaction with Pre-Pregnancy BMI is a Pharmacogenetic Marker for the Initiation of Antenatal Insulin Therapy in Gestational Diabetes Mellitus
Source: Int J Mol Sci. 2018 Nov 23;19(12):3734. doi: 10.3390/ijms19123734 (PMC6321391; doi:10.3390/ijms19123734)
Supplement: Supplementary file 1 [file ijms-19-03734-s001.zip › supplementary material/ijms-379908-Table S1.pdf]

SUPPLEMENTARY TABLE 1:

No of patients with GDM	Treatment modality in the pre-pregnancy BMI defined subgroup	*MTNR1B*rs10830963 genotype distribution in GDM patients with pre-pregnancy BMI < 27 kg/m^2			Risk (*G*) allele frequency
122	**All**	***CC***	***CG***	***GG***	**-**
11	**AIT**	2	8	1	0.45
111	**Non-pharmacological treatment only**	45	51	15	0.36
**No of patients with GDM**	**Treatment modality in the pre-pregnancy BMI defined subgroup**	**MTNR1Brs10830963 genotype distribution in GDM patients with pre-pregnancy BMI ≥ 27 kg/m2**			**Risk (G) allele frequency**
89	**All**	***CC***	***CG***	***GG***	**-**
20	**AIT**	6	9	5	0.48
69	**Non-pharmacological treatment only**	35	28	6	0.29
**No of patients with GDM**	**Treatment modality in the pre-pregnancy BMI defined subgroup**	**MTNR1Brs10830963 genotype distribution in GDM patients with pre-pregnancy BMI < 29 kg/m^2**			**Risk (G) allele frequency**
148	**All**	***CC***	***CG***	***GG***	**-**
15	**AIT**	5	8	2	0.40
133	**Non-pharmacological treatment only**	55	60	18	0.36
**No of patients with GDM**	**Treatment modality in the pre-pregnancy BMI defined subgroup**	***MTNR1B*rs10830963 genotype distribution in GDM patients with pre-pregnancy BMI ≥ 29 kg/m^2**			**Risk (*G*) allele frequency**
63	**All**	***CC***	***CG***	***GG***	**-**
16	**AIT**	3	9	4	0.53
47	**Non-pharmacological treatment only**	25	19	3	0.26

The *MTNR1B* rs10830963 genotype distributions and the number of GDM subjects in the AIT / non-AIT treatment modality subgroups according to the pre-pregnancy BMI cutoffs of 27 kg/m^2 and 29 kg/m^2.
Genotypes with the *risk allele G* are outlined in red colour.
