# Supplementary figures and images for: The MTNR1B rs10830963 Variant in Interaction with Pre-Pregnancy BMI is a Pharmacogenetic Marker for the Initiation of Antenatal Insulin Therapy in Gestational Diabetes Mellitus
Source: Int J Mol Sci. 2018 Nov 23;19(12):3734. doi: 10.3390/ijms19123734 (PMC6321391; doi:10.3390/ijms19123734)

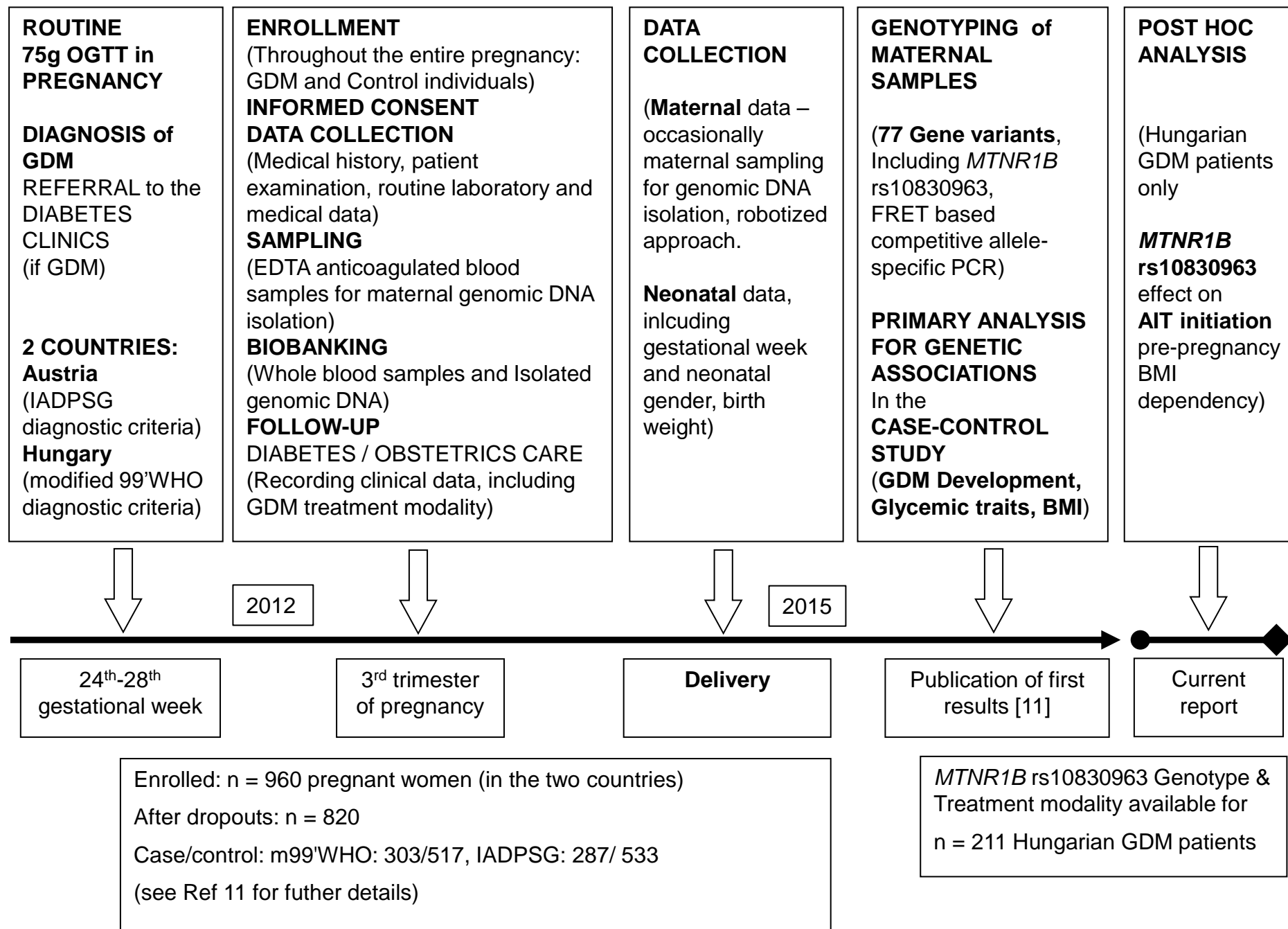

**Flow chart for the original case-control genetic study [11] and for this post hoc analysis**

Supplement: Supplementary file 1 [file ijms-19-03734-s001.zip › supplementary material/ijms-379908-Figure S1.pdf]
